# Pathological Fracture of the Femur Following Chronic Osteomyelitis: A Case Report of This Rare Presentation in Adults

**DOI:** 10.7759/cureus.59733

**Published:** 2024-05-06

**Authors:** Jonathan Botterill, Soubhik Ghosh, Arun Bhaskaran

**Affiliations:** 1 Trauma and Orthopaedics, Queen Elizabeth Hospital, London, GBR

**Keywords:** colovesical fistula, infection, thigh abscess, pathological fracture, osteomyelitis

## Abstract

Chronic osteomyelitis is a common presentation in orthopaedics, particularly in the context of deep abscesses. Fractures related to osteomyelitis can be seen in children but are rare in adults. We present the rare case of a pathological fracture related to osteomyelitis in a 53-year-old warehouse worker who was previously fit and well. He presented with a right-sided thigh abscess and after initial surgical management, further imaging detailed osteomyelitis that was treated with suppressive antimicrobial therapy. Seven months after discharge, the patient presented to our emergency department with a right-sided proximal fracture of the femur. Retrospectively, we were able to localise the fracture site to the site of the previous cloaca from the osteomyelitis. We discuss whether prophylactic fixation is required for patients with extensive cloaca following a chronic fracture.

## Introduction

We present a pathological fracture of the femur in a previously fit and well gentleman presenting with chronic osteomyelitis (COM) and an associated colovesical fistula. The patient developed a right-sided recurrent thigh abscess, resulting in osteomyelitis of the femur on the affected side.

A pathological fracture secondary to osteomyelitis is a rare but recognised complication [1]. Healthy bone is often weakened in the context of COM for the following reasons. Firstly, a subperiosteal abscess elevates the periosteum, reducing blood supply to the underlying bone. The reduced perfusion leads to bone necrosis and then an involucurum is formed from local bone deposited by osteoblasts [2]. This disrupts the structure and force resistance of normal laminar bone. A possible secondary mechanism of weakened bone in COM includes the release of degradative enzymes from neutrophils [3]. Osteoclastic resorption, neutrophilic degradative enzymes and bacterial enzymes weaken the bony architecture, predisposing individuals to the risk of pathological fractures.

## Case presentation

The patient presented to the emergency department with an increase in swelling in his right thigh for the past two weeks with progressively worsening pain, and he was now unable to walk. The patient was a 53-year-old male who worked in a warehouse with a background of mild diverticulitis. On presentation, his C-reactive (CRP) was 462 mg/L, with a white cell count of 40 x109 and a neutrophil count of 36 x109. The following day, an ultrasound scan of the thigh demonstrated a large fluid collection in the quadriceps and adductor muscles spreading to the gluteals on the affected side. This was aspirated rather than formally washed out, as the patient was not starved, and 20 ml of feculent pus was drained. A computerised tomography scan of the chest, abdomen and pelvis on admission had also noted a colovesical fistula incidentally.

Following the aspiration, the patient underwent a number of washouts for source control, with six in total over the next month. Next, microbiology isolated Candida albicans, Enterococcus faecium and Aspergillus niger. Following the final washout, the wound was closed, however, it was noted that one of the four heads of the quadriceps was fully lysed and destroyed by the infection. The colovesical fistula was discussed with the general surgery team as a possible source for the abscess, however, no fistula between the anterior thigh and bowel was demonstrated with a gastrograffin study. Further inpatient evaluation with colonoscopy demonstrated diverticulitis at the site of the fistula, but no obvious fistula connecting to the thigh. The final washout was completed a month later, with the closure of the wound alongside antimicrobial therapy.

**Figure 1:**
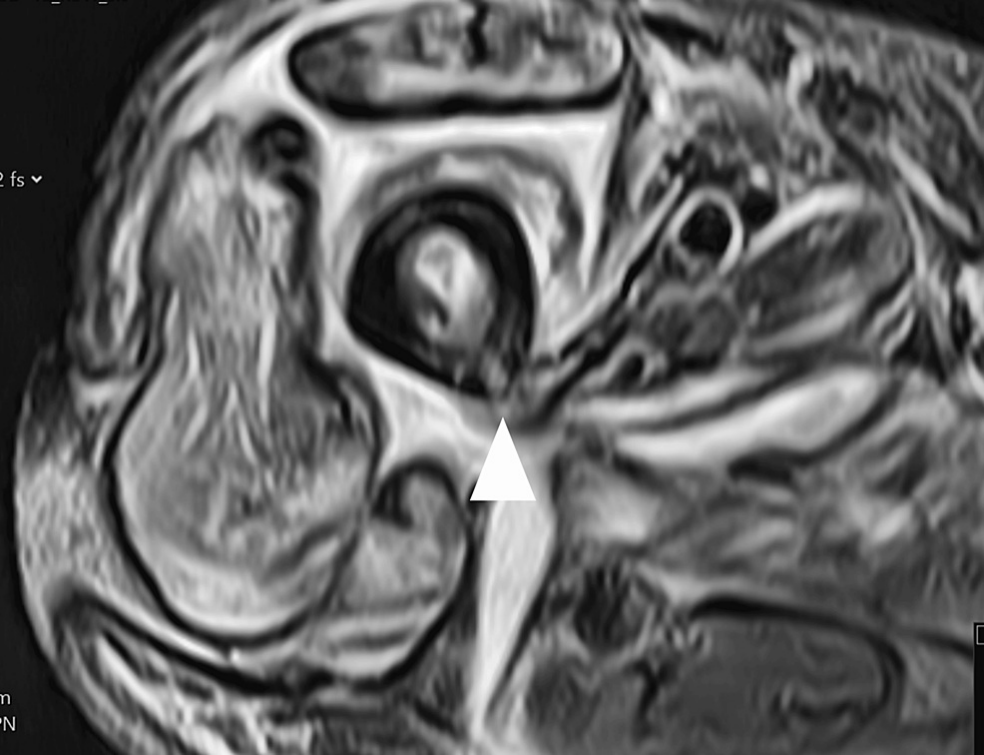
A T2-weighted axial slice of the right thigh The report noted hyperintensity in the anterior compartment where the majority of the fluid was. Highlighted are osteomyelitis-related changes. The high T2 signal in the bone marrow represents femoral canal necrosis.

**Figure 2:**
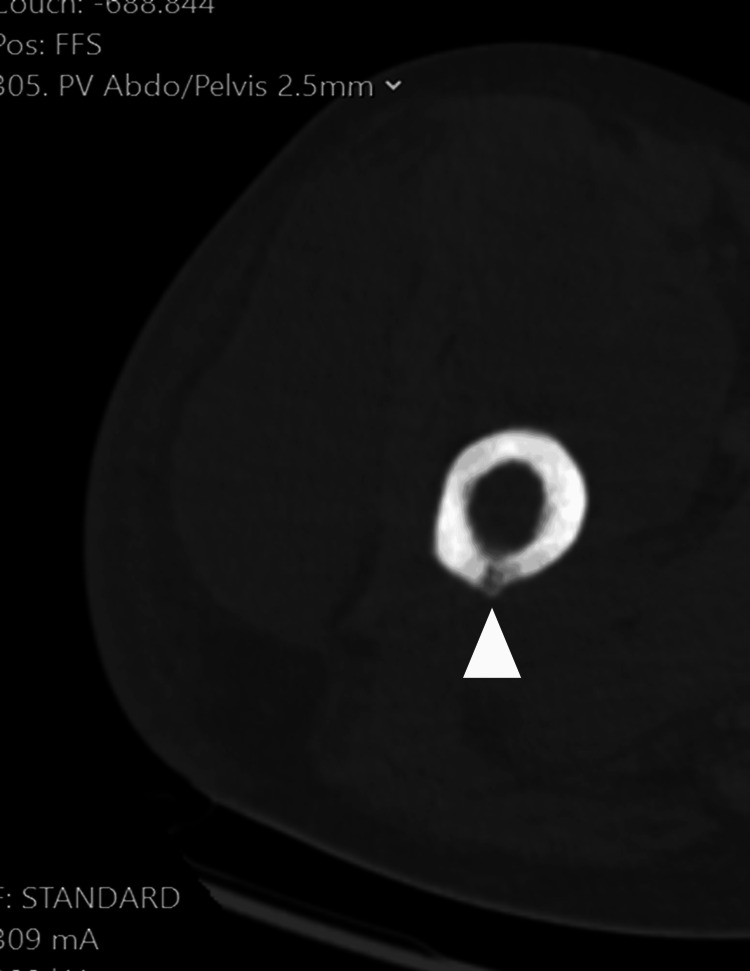
An axial slice of a plain CT obtained on admission to the emergency department On a retrospective review of the images with the radiology department, a cloaca can be seen in the posterior aspect of the proximal right thigh as a cortical disruption.

**Figure 3:**
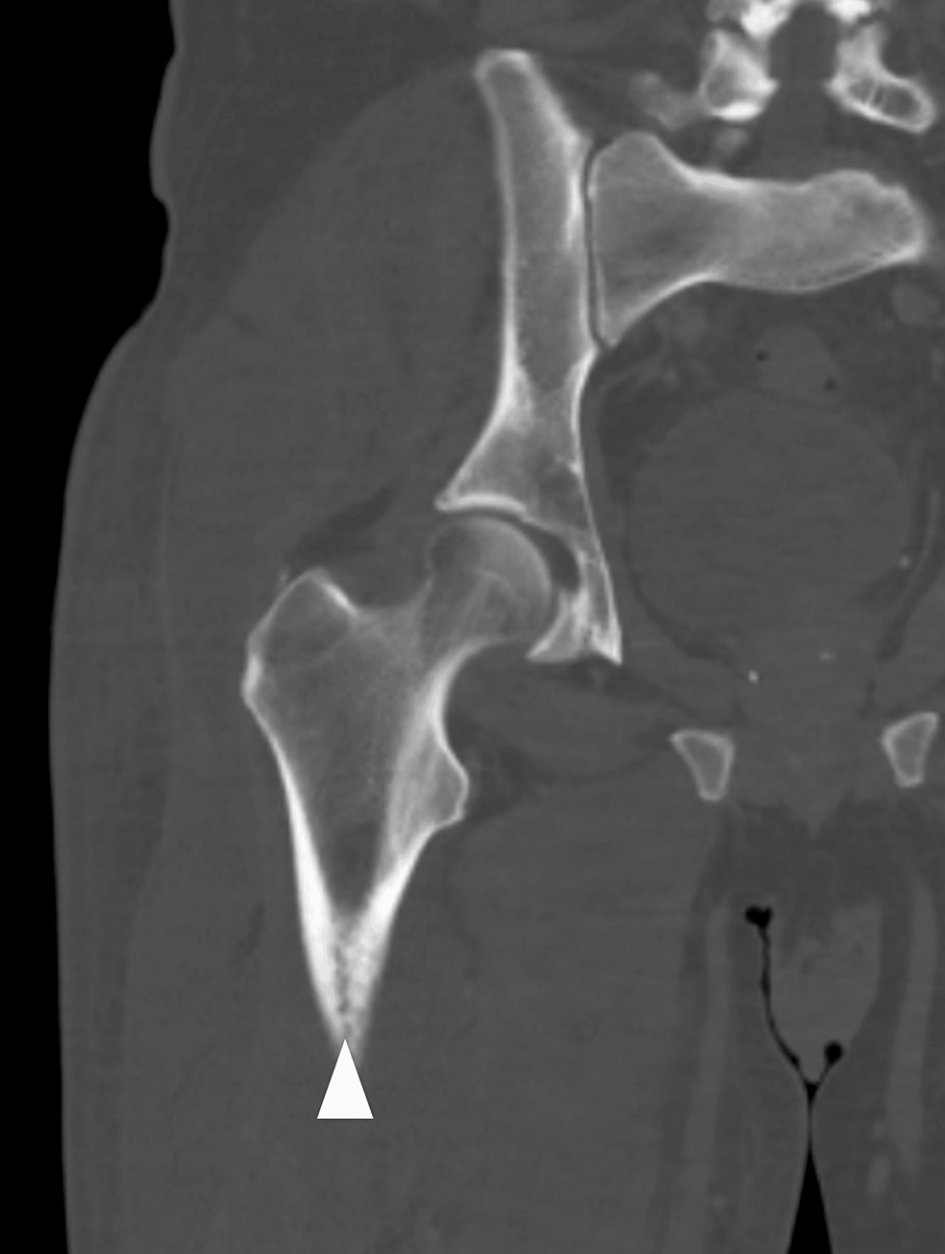
A coronal section of the right femur demonstrating the cloaca

Magnetic resonance imaging performed two weeks into admission demonstrated necrosis of the femoral bone marrow along with adductor and gluteal muscle necrosis and fluid in the fascial planes (Figure 1). On a review of previous CT imaging taken on admission, a small cloaca was noted in the posterior aspect of the proximal femur around the location of the osteomyelitis seen on CT (Figures 2, 3). A discussion between the department and the regional bone infection unit advised a femoral reaming to assess for associated microbiology. This was performed a month after the initial presentation and the wound was closed for the final washout. The wound was reopened to allow for femoral canal debridement, and samples from this produced a heavy growth of E. coli. CT angiogram of the right lower limb was then performed, which radiologically demonstrated COM; this was the first diagnosis as seen in Figure 1. The patient was discharged following a further two months of IV antibiotics for treatment of the bone infection.

**Figure 4:**
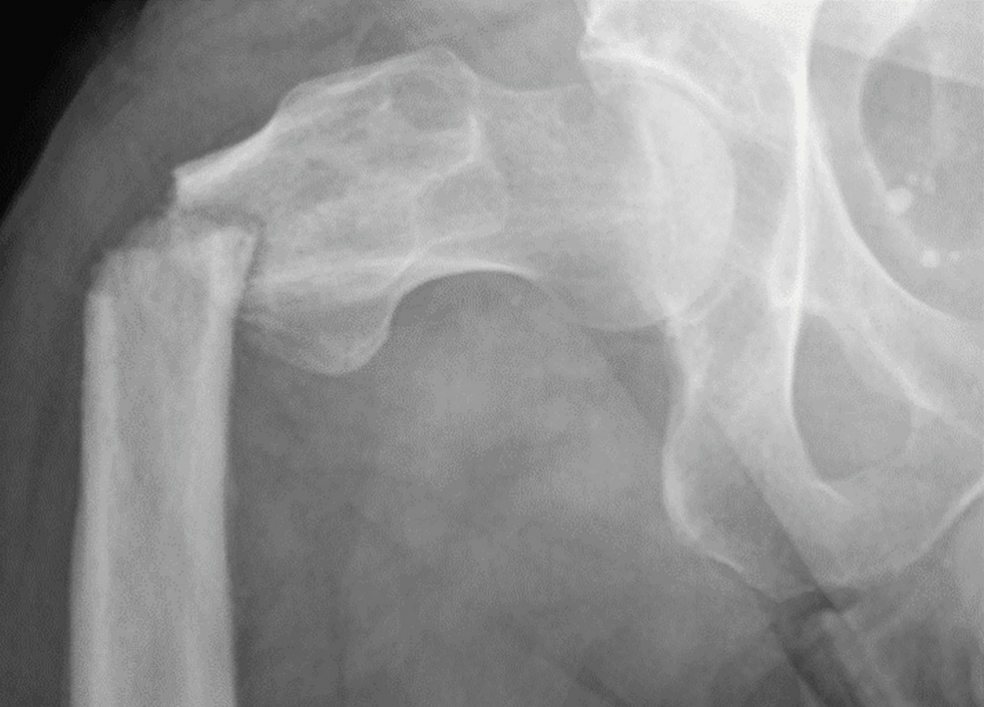
An AP radiograph taken on admission when the patient returned with the proximal femoral fracture AP: anteroposterior

Seven months following discharge, the patient presented to the emergency department with severe pain following a fall on to the right leg. X-rays demonstrated a fracture of the proximal femur (Figure 4).

This was fixed four days later with a long intramedullary nail. Cultures intraoperatively again grew E. coli. The patient was discharged on IV ceftriaxone and oral antifungals delivered in our ambulatory unit for a further month, to complete a total of three months of IV antibiotics with a switch to ertapenem. This was the suppressive treatment suggested by our microbiology team. The patient struggled to make their appointment at the bone infection unit given transport issues. Six months after the operation, there was satisfactory callous formation and healing.

## Discussion

MRI is an important modality to consider to rule out osteomyelitis in any patient presenting with an abscess of the thigh, as any deep soft tissue infection can spread and cause osteomyelitis [4].

This case was of note given its uncommon microbiology, namely, the enteric flora as causative organisms. The vast majority of osteomyelitis is caused by Staphylococcus aureus, which is the major causative organism in haematogenous spread, with gram-negative bacilli causing anywhere from 3-11% of osteomyelitis infections [5]. The enteric organisms and noted enteric fistula led the MDT (multidisciplinary team, comprising orthopaedic surgeons, radiologists and infectious disease doctors) to believe the source was from this fistula. It was only after the gastrograffin study undertaken by the general surgical team demonstrated no continuity between the bowel and thigh that it was believed to be a haematogenous spread.

On review of the images, we were able to localise the fracture site to the previous cloaca sites. Figure 3 demonstrates the cloaca in the coronal view with associated cortical disruption in the proximal femur. Figure 4 demonstrates the displaced fracture occurring in this area, implicating the cloaca and associated osteomyelitis weakened the bone leading to this pathological fracture.

This case also raises the question as to whether a prophylactic scoring system for chronic osteomyelitis should be considered, similar to the scoring system often used in cancer (Mirel’s score). Mirel’s score takes into account the location of the lesion, the associated pain and the nature of the lesion. Fixation related to osteomyelitis is seen in children via the Masquelet technique, however, no such approach appears to be in use for adults [6].

## Conclusions

This case highlights the importance of an early diagnosis of thigh abscesses and close follow-up to ensure the patient does not develop femoral osteomyelitis, due to the associated risk of pathological fracture. This case also raises the question as to whether a prophylactic scoring system needs to be developed for osteomyelitis.
